# Relating protein crystal structure to ligand-binding thermodynamics

**DOI:** 10.1107/S2053230X22011244

**Published:** 2022-11-28

**Authors:** John R. Helliwell

**Affiliations:** a Department of Chemistry, University of Manchester, Manchester M13 9PL, United Kingdom; Centro Nacional de Biotecnología – CSIC, Spain

**Keywords:** protein ligand binding, thermodynamics and structure, lectins, saccharides

## Abstract

An important interface between biophysical chemistry and biological crystal structures involves whether it is possible to relate experimental calorimetry measurements of protein ligand binding to 3D structures. This has proved to be challenging. The probes of the structure of matter, namely X-rays, neutrons and electrons, have challenges of one type or another in their use. This article focuses on saccharide binding to lectins as a theme, yet after 25 years or so it is still a work in progress to connect 3D structure to binding energies. Whilst this study involved one type of protein (lectins) and one class of ligand (monosaccharides), *i.e.* it was specific, it was of general importance, as measured for instance by its wide impact. The impetus for writing this update now, as a *Scientific Comment*, is that a breakthrough in neutron crystal structure determinations of saccharide-bound lectins has been achieved. It is suggested here that this new research from neutron protein crystallography could improve, *i.e.* reduce, the errors in the estimated binding energies.

## Abstract 2

Is it possible to relate experimental calorimetry measurements of protein ligand binding to 3D structures?

An important interface between biological crystallography and biophysical chemistry involves the question of whether a 3D structure can be quantitatively linked to its thermodynamics. It is 24 years since an article was published linking the protein–saccharide complexes of concanavalin A with mannoside or glucoside to their binding data from isothermal calorimetry (Bradbrook *et al.*, 1998 ▸). To secure this link, molecular-dynamics studies of the crystal structures of concanavalin A bound to α-methyl mannoside or glucoside were required as starting points. As validation, the atomic displacement parameters from the molecular-dynamics studies and the X-ray crystal structures showed reasonable agreement. However, the uncertainty values in the Gibbs free energies of binding of these two saccharides were too large to ensure a precise confirmation from the structures. That said, the chance to observe fleeting hydrogen bonds during the time simulations did improve the agreement. The structural details of this study (Bradbrook *et al.*, 1998 ▸) are depicted in Fig. 1 ▸.

This study (Bradbrook *et al.*, 1998 ▸) combined, for the first time, state-of-the-art (at the time) protein crystallography with state-of-the-art (at the time) theoretical chemistry. Whilst our study involved one type of protein (lectins) and one class of ligand (monosaccharides), *i.e.* it was specific, it was of general importance, as measured for instance by its gaining over 100 citations.

Bradbrook *et al.* (1998 ▸) developed a ‘master equation’

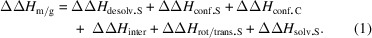

In this equation, we considered the difference in binding enthalpy for mannoside (m) and glucoside (g), on binding to concanavalin A (C), to be due to a combination of the following terms in the equation.

(i) A difference in the perturbation of water around the sugars (S) on complexation with the protein (ΔΔ*H*_desolv.S_).
(ii) A difference in the changes in configurational enthalpy for the sugar (S) and/or the concanavalin A protein (C) (ΔΔ*H*_conf.S_, ΔΔ*H*_conf.C_).
(iii) Different interactions within the mannoside complex with the protein compared with that of glucoside with the protein (ΔΔ*H*_inter_), which are provided by the crystal structures.
(iv) A dynamical motion of the sugars within the active site leading to different average interactions for the two sugars (ΔΔ*H*_inter_, here taken as the average over an ensemble, and ΔΔ*H*_rot/trans.S_, the contribution of rotation and translation to the enthalpy difference).
(v) A difference in the solvation of the complexes (ΔΔ*H*_solv.CS_).

The term (iv) above that considered a possible dynamical motion of the sugars within the active site, leading to different interactions for the two sugars, proved to be pivotal. The total length of time simulated was around 0.5 ns. The simulation time step, each with a new calculated structure, was 0.5 ps. The complete simulation comprised 500 sets of new protein with sugar coordinates. Analysis of the molecular-dynamics sequences revealed transiently forming hydrogen bonds between atoms of one of the sugars compared with the other. We had thus managed to reveal a dynamical picture of the protein and its sugar interactions. However, the study had its limitations. Most obvious was the fact that X-ray crystallo­graphy did not reveal the H atoms on the sugars or in the binding site on the protein. Secondly, the time lengths of the molecular-dynamics simulations were quite short. That said, thus study (Bradbrook *et al.*, 1998 ▸), which was expanded on in Bradbrook *et al.* (2000 ▸), considered the issue of enthalpy–entropy compensation hampering ligand design. Such efforts try to obtain tighter binding, but this is incorrect. The way to design ligands then, as advocated in Bradbrook *et al.* (1998 ▸), is to obtain binding that is a little less tight but with more potential configurations. Gail Bradbrook (personal communication) vividly describes this:

think about the ligand dancing within a binding site – like a form of jiving or flutter binding – so it can have both high enthalpy good binding when it is in a particular configuration, and high entropy because it moves around as entropy implies, but in that movement a different amino acid grabs hold of the ligand.

We perceive that this was a groundbreaking observation to describe protein–ligand interactions as ‘ligand dancing’, thereby differing from either the classical lock-and-key concept of Emil Fischer or the induced-fit concept of Daniel Koshland.

In terms of crystallography, great strides have been made in X-ray and neutron central facilities over the past decades, greatly expanding the pace of protein crystal structure determination. Part of this expansion has relied on cryo-crystallo­graphy to protect protein crystals from damage from ultra-intense synchrotron X-ray radiation beams. I use the term ultra-intense to distinguish second-generation synchrotron facilities from the incomparably superior beam intensities at third-generation synchrotron sources by factors of >100 or more. It has been found that there is a price to using cryo-temperatures, which is the growing evidence that there is intrinsic plasticity of a protein such that there can be changes in the protein structure between room temperature and 100 K and in its associated hydration, namely its bound water structure. An early paper noting these temperature-driven differences was published by us in *Faraday Transactions* (Deacon *et al.*, 1997 ▸). These differences were elaborated on by Halle (2004 ▸). There is thus a growing effort to determine room-temperature protein crystal structures. Neutron protein crystallography has an intrinsic advantage here as it not only determines protein crystal structures that are complete with hydrogens (as deuteriums) but also, as a nondamaging probe, can work routinely at room temperature. The crystal structures worked with in Bradbrook *et al.* (1998 ▸) were based on X-ray diffraction data measured at room temperature, which is biologically relevant to the case of plants and a plant protein such as jack bean concanavalin A.

Let us reflect more deeply on this point of the temperature of the biological organism from which a protein of interest is being studied, given that the overarching aim is to determine its structure and function. Plants grow, for example, in our gardens at ambient temperature, and clearly this is not the same as mammals such as ourselves with a body temperature of 37°C. This theme of measuring protein crystal diffraction data at the temperature of the functioning organism I suggest to be important. Thus, mammalian proteins should be further investigated at their working temperature of 37°C. To emphasize the point further, I mention that thermophiles should also be studied at their working temperatures. These latter studies will perhaps most likely be performed in solution using methods such as NMR rather than protein crystallo­graphy. The case of hyperthermophilic proteins is even more challenging for structural studies, as even NMR would find superheated steam a challenging sample state in which to measure resonances for a protein. Overall, if our aim is to study structure and function then I commend that it is necessary to determine such a protein structure at its functioning temperature. As a protein ligand theme with which to study the efficacy of binding-energy calculations, a plant protein such as jack bean concanavalin A is therefore a good place to start. Moreover, if we can successfully obtain accurate calculations of protein ligand-binding energies, then the more challenging cases of elevated-temperature organisms could perhaps use such calculations to corroborate structural studies, which may well have to be made well away from the working temperature of a protein, with hyperthermophiles being the most challenging to physical methods as described above.

Carbohydrate–protein interactions have been extensively reviewed by Pérez & Tvaroška (2014 ▸), including descriptions of the various approaches that have been undertaken to investigate the relationship between 3D structure and the thermodynamics of binding. Our study (Bradbrook *et al.*, 1998 ▸) proved to be the simplest conceptually, being a study of two closely similar monosaccharides, rather than being based on oligosaccharides (Bradbrook *et al.*, 2000 ▸; Bryce *et al.*, 2001 ▸). Also, it involved ligand binding rather than enzymatic catalysis, which throws up additional complications of a reactant changing to a product. The simplest extension of the concept of Bradbrook *et al.* (1998 ▸) was to compare a di­saccharide and a monosaccharide, rather than two monosaccharides, and this was undertaken by Bradbrook *et al.* (2000 ▸). However, the second saccharide in the disaccharide, most distal from the protein, has a wide range of positions in the molecular-dynamics simulation based on the crystal structure (Fig. 2 ▸).

The field of neutron macromolecular crystallography, as mentioned above, has made great strides (see Helliwell, 2020 ▸, for a recent summary). It provides details of protonation states, hydrogen bonding and orientation of water molecules that are impossible to obtain by any other method for crystals diffracting to better than 2.5 Å resolution. The advent of large-area neutron-sensitive detectors as well as of deuteration microbiology for full deuteration of the protein, an expansion of the global suite of instruments and finally the extension of fully validated Laue diffraction data-processing software from the Daresbury synchrotron laboratory to electronic detector data from the European Synchrotron Radiation Facility (Nieh *et al.*, 1999 ▸) has enabled numerous neutron protein crystallography based structural biochemistry studies which were otherwise at an impasse for X-ray protein crystallography or NMR. Within the specific theme of concanavalin A saccharides, Gerlits *et al.* (2017 ▸) determined a room-temperature neutron crystal structure of this legume lectin in complex with the disaccharide mannobiose. The neutron structure afforded direct visualization of the hydrogen bonding between the protein and ligand, showing that the ligand is able to alter both the protonation states and interactions for residues located close to and distant from the binding site. The most recent report in the overall protein–saccharide binding theme is that by Shukla *et al.* (2022 ▸), who reported a room-temperature neutron crystal structure of maltodextrin periplasmic-binding protein (PBP) in complex with an oligosaccharide. Indeed, this is the first neutron crystal structure from the PBP superfamily, and it unambiguously determines the nature and orientation of the hydrogen-bonding and water-mediated interactions involved in stabil­izing a tetrasaccharide in the binding site.

A distinctive difficulty that has had to be surmounted is incorporating the production and the use of fully deuterated ligands in general, and saccharides in particular, into the above repertoire of developments. A recent breakthrough in experimental crystal structures came from studies of the fucose-specific lectins PLL and LecB from *Photorhabdus laumondii* and *Pseudomonas aeruginosa*, respectively. These were produced in perdeuterated forms and crystallized with a perdeuterated monosaccharide, l-fucose, using genetically modified strains of *Escherichia coli*. Three neutron crystal structures have been solved. These were specifically crystal structures of PLL from *P. laumondii* in both apo and ligand-bound forms and a crystal structure of LecB from *P. aeruginosa* in complex with perdeuterated fucose. Thus, these studies provided the first experimental determinations of the directionality of the fucose hydroxyl groups and the protonation states of acidic residues in the carbohydrate-binding site of LecB from the human pathogen *P. aeruginosa* (Gajdos *et al.*, 2021 ▸, 2022 ▸). The neutron crystal structures included apo and monosaccharide-bound forms; for an example of the fine details that can be seen in the nuclear density map, see Fig. 3 ▸. The prospects are bright for revisiting the study of Bradbrook *et al.* (1998 ▸), or its equivalent, but with neutron protein crystal structures.

There is an interesting extrapolation of these basic scientific studies to the case of structure-based drug design. In *in vivo* and *in vitro* laboratory studies, the working temperature of the former (37°C) and the typical working laboratory temperature of the latter (20°C) can be investigated using crystallography. Does this temperature difference matter for the objectives of medicine design? For basic science to help, we need to investigate protein crystal structures, and their ligand binding, at 37°C. This is beginning to be within reach, with accessible facilities at synchrotrons and X-ray free-electron lasers (see, for example, Huang *et al.*, 2022 ▸). I suggest that the extensive book on the topic of protein–ligand interactions written in 2003 (Böhm & Schneider, 2003 ▸) is ripe for an update.

To sum up, I commend that it is important to perform more neutron protein crystallography case studies on, for example, protein–saccharide complexes to try and bridge the fields of protein structural science and protein ligand-binding energetics. If we are to make this bridge, then the estimated binding energies from calculation must be more precise than those achieved by Bradbrook *et al.* (1998 ▸). Neutron protein crystallography is a promising approach. Within this theme, an even more ambitious research program would be to measure neutron protein crystallography diffraction data sets for one or more model systems at different temperatures so that protein ligand-binding energies, enthalpies and entropies can be more reliably estimated.

## Figures and Tables

**Figure 1:**
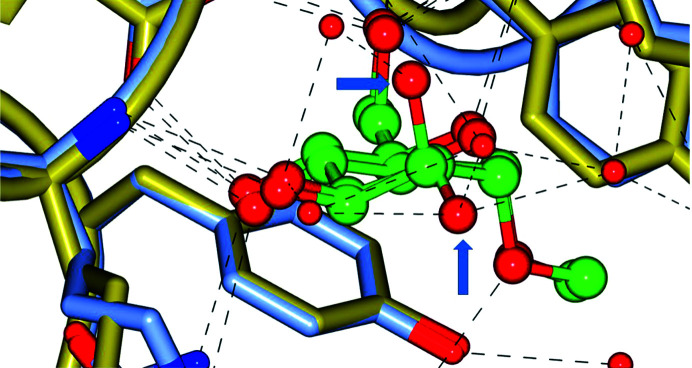
View of the sugar-binding site of concanavalin A studied by X-ray crystallography enlarged to view the two sugars superimposed. The smaller red spheres are the O atoms of bound water molecules. The H atoms are not displayed as these were not experimentally determined. The two crystal structures have been deposited in the Protein Data Bank as entries 1gic (the protein with glucoside) and 5cna (the protein with mannoside; Naismith *et al.*, 1994 ▸). The dashed lines are putative hydrogen bonds. The blue arrows point out the key difference between the mannoside and glucoside O2 atom orientations, *i.e.* the mannoside orientation is vertical. This figure was prepared using *CCP*4*mg* (McNicholas *et al.*, 2011 ▸).

**Figure 2:**
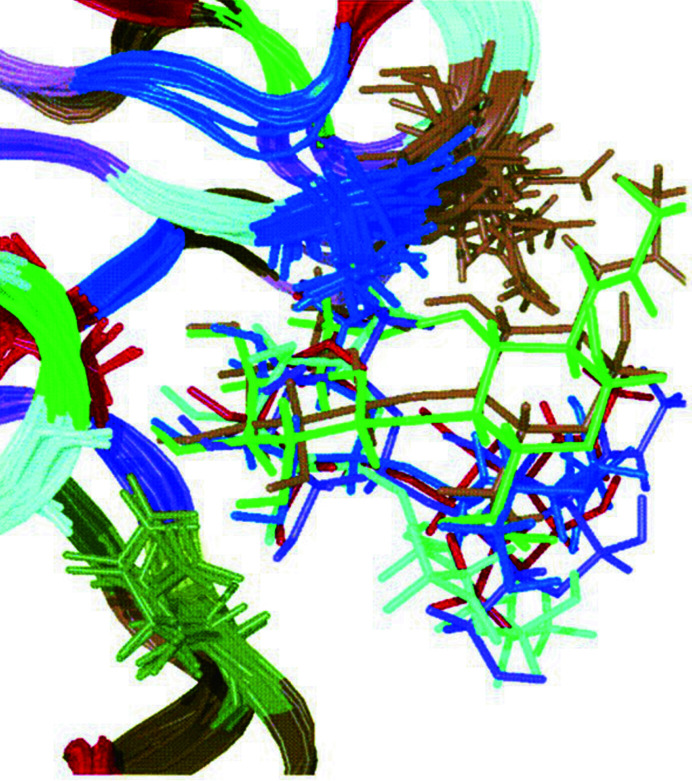
Snapshot structures (each with the sugar in a different colour) taken from the molecular-dynamics simulation of the *N*-acetyllactosamine complex reported by Bradbrook *et al.* (2000 ▸). Reproduced with permission from Wiley and Dr Bradbrook.

**Figure 3:**
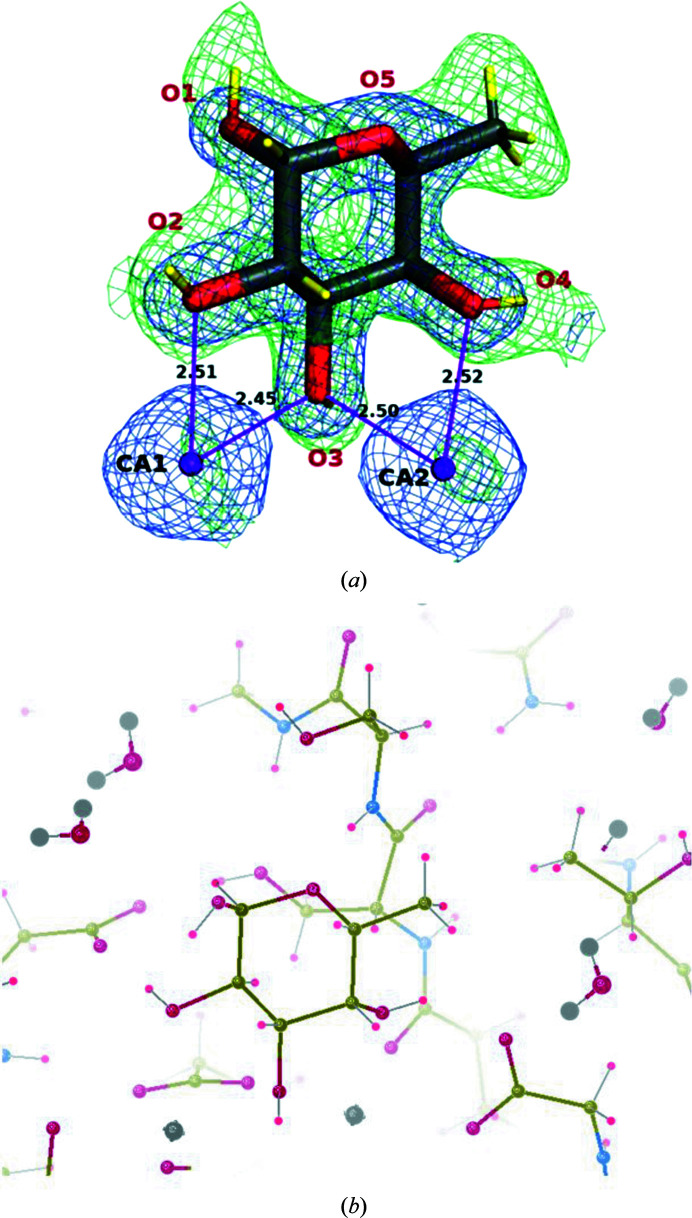
(*a*) Perdeuterated l-fucose coordinating two Ca atoms in the binding site of LecB (Gajdos *et al.*, 2022 ▸). Calcium ions are shown as purple spheres, metal coordination as solid purple lines (with lengths labelled in Å) and D atoms as yellow sticks. 2*mF*_o_ − *DF*_c_ neutron scattering-length density (green mesh) and 2*mF*_o_ − *DF*_c_ electron density (blue mesh) are contoured at 1.4σ and 2.2σ, respectively. Reproduced from Gajdos *et al.* (2022 ▸). (*b*) Using the same orientation as in (*a*), this shows fucose bound to the protein with the H atoms now determined experimentally (PDB entry 7prg; this figure was obtained using *Coot* (Emsley *et al.*, 2010 ▸).

## References

[bb1] Böhm, H.-J. & Schneider, G. (2003). Editors. *Protein–Ligand Interactions: From Molecular Recognition to Drug Design.* Weinheim: Wiley-VCH.

[bb3] Bradbrook, G. M., Forshaw, J. R. & Pérez, S. (2000). *Eur. J. Biochem.* **267**, 4545–4555. 10.1046/j.1432-1327.2000.01505.x 10880979

[bb2] Bradbrook, G. M., Gleichmann, T., Harrop, S. J., Habash, J., Raftery, J., Kalb (Gilboa), J., Yariv, J., Hillier, I. H. & Helliwell, J. R. (1998). *Faraday Trans.* **94**, 1603–1611.

[bb4] Bryce, R. A., Hillier, I. H. & Naismith, J. H. (2001). *Biophys. J.* **81**, 1373–1388. 10.1016/S0006-3495(01)75793-1 PMC1301617 11509352

[bb5] Deacon, A., Gleichmann, T., Kalb (Gilboa), A. J., Price, H., Raftery, J., Bradbrook, G., Yariv, J. & Helliwell, J. R. (1997). *Faraday Trans.* **93**, 4305–4312.

[bb6] Emsley, P., Lohkamp, B., Scott, W. G. & Cowtan, K. (2010). *Acta Cryst.* D**66**, 486–501. 10.1107/S0907444910007493 PMC2852313 20383002

[bb7] Gajdos, L., Blakeley, M. P., Haertlein, M., Forsyth, V. T., Devos, J. M. & Imberty, A. (2022). *Nat. Commun.* **13**, 194. 10.1038/s41467-021-27871-8 PMC8752737 35017516

[bb8] Gajdos, L., Blakeley, M. P., Kumar, A., Wimmerová, M., Haertlein, M., Forsyth, V. T., Imberty, A. & Devos, J. M. (2021). *Structure*, **29**, 1003–1013. 10.1016/j.str.2021.03.003 33765407

[bb9] Gerlits, O. O., Coates, L., Woods, R. J. & Kovalevsky, A. (2017). *Biochemistry*, **56**, 4747–4750. 10.1021/acs.biochem.7b00654 PMC5754002 28846383

[bb10] Halle, B. (2004). *Proc. Natl Acad. Sci. USA*, **101**, 4793–4798. 10.1073/pnas.0308315101 PMC387327 15051877

[bb11] Helliwell, J. R. (2020). *Methods Enzymol.* **634**, 1–19. 10.1016/bs.mie.2020.01.006 32093828

[bb12] Huang, C.-Y., Aumonier, S., Engilberge, S., Eris, D., Smith, K. M. L., Leonarski, F., Wojdyla, J. A., Beale, J. H., Buntschu, D., Pauluhn, A., Sharpe, M. E., Metz, A., Olieric, V. & Wang, M. (2022). *Acta Cryst.* D**78**, 964–974. 10.1107/S205979832200612X PMC9344481 35916221

[bb13] McNicholas, S., Potterton, E., Wilson, K. S. & Noble, M. E. M. (2011). *Acta Cryst.* D**67**, 386–394. 10.1107/S0907444911007281 PMC3069754 21460457

[bb14] Naismith, J. H., Emmerich, C., Habash, J., Harrop, S. J., Helliwell, J. R., Hunter, W. N., Raftery, J., Kalb (Gilboa), A. J. & Yariv, J. (1994). *Acta Cryst.* D**50**, 847–858. 10.1107/S0907444994005287 15299352

[bb15] Nieh, Y. P., Raftery, J., Weisgerber, S., Habash, J., Schotte, F., Ursby, T., Wulff, M., Hädener, A., Campbell, J. W., Hao, Q. & Helliwell, J. R. (1999). *J. Synchrotron Rad.* **6**, 995–1006.

[bb16] Pérez, S. & Tvaroška, I. (2014). *Adv. Carbohydr. Chem. Biochem.* **71**, 9–136. 10.1016/B978-0-12-800128-8.00001-7 25480504

[bb17] Shukla, S., Myles, D. A. & Cuneo, M. J. (2022). *Sci. Rep.* **12**, 17647. 10.1038/s41598-022-20542-8 PMC9587032 36271099

